# Gender Differences in Type 1 Diabetes Management and Mental Health Burden: Findings from a National Survey in Saudi Arabia

**DOI:** 10.3390/jcm14165777

**Published:** 2025-08-15

**Authors:** Abdullah M. Alguwaihes

**Affiliations:** Department of Internal Medicine, College of Medicine, King Saud University, Riyadh 11451, Saudi Arabia; aalguwaihes@ksu.edu.sa

**Keywords:** type 1 diabetes, mental health, PHQ-9, Saudi Arabia, depression, anxiety

## Abstract

**Background:** T1D is generally associated with increased psychological burden, but evidence from Middle Eastern populations remains scarce. The present study assessed the gender differences in management, prevalence, and risk factors of perceived depression and anxiety among people with T1D in Saudi Arabia. **Methods:** A cross-sectional online survey was conducted among people withT1D across Saudi Arabia to collect demographic, clinical, and diabetes management data. Perceived depression and anxiety symptoms were assessed using a validated questionnaire. Multinomial logistic regression was used to identify risk factors of severe depression and anxiety. **Results:** Among 1073 PwT1D (303 males and 770 females), perceived depressive and anxiety symptoms were highly prevalent. Females had higher perceived anxiety compared with males (*p* = 0.003). Age- and BMI-adjusted regression analysis showed that, overall, higher income (*p* = 0.008), no neuropathy (*p* = 0.002), above-average benefit from the diabetes education clinic (*p* = 0.02), practicing carbohydrate counting (*p* = 0.002), and HbA1c < 7.0% (*p* = 0.01) were protective against perceived severe depression. Friends with T1D as the preferred education source (odds ratio [OR] = 2.8, *p* = 0.04) and a rejected request for continuous glucose monitoring (CGM) (OR = 1.88, *p* = 0.02) or insulin pump (OR = 2.8, *p* = 0.001) were significant risk factors. Perceived severe anxiety was associated with insulin pump rejection (OR = 2.4, *p* < 0.001) and self-reading as the preferred education source (OR = 2.0, *p* = 0.03). Being male (*p* = 0.02), no neuropathy (*p* = 0.01), practicing carbohydrate counting (*p* < 0.001), and HbA1c < 7.0% (*p* = 0.001) were protective. **Conclusions:** Symptoms of depression and anxiety are highly prevalent among people with T1D in Saudi Arabia, with females and socioeconomically disadvantaged individuals at greater risk. The findings highlight an urgent need for integrated mental health support within diabetes care and improved access to resources.

## 1. Introduction

Type 1 diabetes (T1D) is a form of chronic autoimmune disorder that requires lifelong insulin therapy and ongoing self-management for survival [1]. The psychological burden of managing T1D can be substantial, often leading to depression and anxiety, which, in turn, negatively impact glycemic control and overall quality of life [2,3,4]. The prevalence and predictors of mental health issues in T1D are relatively well known and have been extensively studied worldwide, with depression, anxiety, and feeding and eating disorders as the most frequently investigated conditions [5]. Globally, the prevalence of depression among young people with T1D is estimated to be 22% based on 109 studies involving more than 52,000 children with T1D or type 2 diabetes (T2D) [6]. Higher rates were observed in girls than in boys (29.7% vs. 19.7%), and in lower to middle-income countries (29.3%) [6].

The psychological burden of T1D can be partially explained through theoretical frameworks such as self-regulation theory, which suggests that individuals must continuously monitor and adapt their behaviors to manage chronic conditions. This often leads to emotional exhaustion when discrepancies between goals and outcomes persist [7]. Coping strategies, including problem-focused (e.g., carbohydrate counting) and emotion-focused (e.g., seeking social support) approaches are critical in alleviating distress. However, maladaptive coping mechanisms, such as reliance on unqualified peer advice, may exacerbate vulnerability to psychological stress [2,3,4]. Emotional awareness and social learning also play an important role in diabetes self-management. For instance, parental sensory processing sensitivity influences children’s attention to emotional cues, which may extend to peer interactions in youth with T1D communities [8]. This framework suggest how insufficient professional support can increase psychological risks in people with type 1 diabetes, particularly in caregiving roles, where stress is closely associated with anxiety [9]. Moreover, gender differences in self-reporting may skew prevalence, as males often under-report psychological distress due to coping styles and atypical symptoms, leading to more severe outcomes [10,11,12]. Recent studies from Saudi Arabia support this, showing elevated anxiety/depression rates in females [13].

In Saudi Arabia, most studies on mental health and diabetes have focused on adults and T2D, with small cohorts concentrated in select regions, limiting generalizability [14,15,16,17]. Across these studies, the prevalence of depression and anxiety consistently approached 50% among patients with T2D based on self-reported data collected using validated assessment tools, such as the Arabic version of the Patient Health Questionnaire-9 (PHQ-9) and the General Anxiety Disorder-7 (GAD-7) questionnaire [18,19].

For people with T1D, there are even fewer studies. One small study performed in the Qassim region (*n* = 148 T1D) showed that severe depression was present in 7.5% of respondents and identified exacerbating factors, which included being female and uncontrolled HbA1c [20]. One larger-scale study in Taif (*n* = 536 T1D; 315 males and 221 females) found that depression and anxiety were prevalent in 68.5% and 30.3% of people with T1D, respectively, and both positively correlated with illness uncertainty measured using the Mishel Uncertainty of Illness Scores [13]. To the best of our knowledge, no study has investigated the overall prevalence of depression and anxiety in Saudi people with T1D. With an increasing incidence of T1D in Saudi Arabia [21], there is a clear gap in local evidence on the mental health of people with T1D in the Saudi population, highlighting the need for larger-scale studies to inform and guide health policy decisions.

Given the cultural and healthcare system differences within ethnic populations, understanding the mental health challenges among Saudi people with T1D is essential to patient-centered care and policy development. This study aimed to assess the gender differences in T1D management and prevalence of perceived depression and anxiety symptoms and to identify risk factors of severe psychological distress in a large cohort of people with T1D in Saudi Arabia.

## 2. Materials and Methods

### 2.1. Study Design and Participants

This cross-sectional study was conducted in Saudi Arabia. Only Saudi people with T1D who were able to completely answer the survey were included in the analysis. This ensured data integrity for multinomial regressions, as incomplete surveys often lacked key variables like PHQ-9/GAD-7 scores. No imputation was used, aligning with cross-sectional design. The questionnaire consisted of three parts: demographic and medical data, the PHQ-9 [18], and the GAD-7 [19]. The questionnaire was hosted on the SurveyMonkey platform and disseminated through multiple channels to ensure a broad and diverse reach; it was shared with people with T1D following visits at diabetes clinics at King Khaled University Hospital, and patients were encouraged to share the questionnaire with their T1D circle. It was also shared in social media groups dedicated to people with T1D and advocacy efforts within the Saudi T1D community. Medical experts with large social media followings in the T1D community helped promote the survey to enhance outreach. Diabetes educators and dietitians from different regions were also invited to distribute the survey link to their patients and share it within their professional and community networks. This multi-pronged strategy was designed to generate a snowball sampling effect, maximizing participant engagement and sample diversity. However, as a convenience method, it resulted in ~48% of participants being from the central region, which, while partially reflective of Saudi demographics [22], may limit representativeness of less populous areas.

The study protocol and procedures conformed to international ethics guidelines. The study was approved by the Institutional Review Board of the College of Medicine, King Saud University, Riyadh, Saudi Arabia (Approval no: E-21-5928, 27 May 2021).

### 2.2. Data Analysis

Data were analyzed using SPSS version 21.0 (IBM, Armonk, NY, USA). Continuous variables are presented as mean ± standard deviation (SD), and categorical variables are presented as frequencies (*n*) and valid percentages (%). Student’s *t*-test was used to compare age and body mass index (BMI) between males and females, whereas chi-squared was used for comparisons of all categorical variables. Multinomial logistic regression analysis was carried out to determine significant risk factors of severe depression and severe anxiety (dependent variables), using gender, education, income, physical activity, diabetes complications, other diseases, diabetes clinic visits, education benefits, education resource preference, advised carbohydrate counting, carbohydrate counting practices, dietitian clinic visits, last HbA1c, insurance, rejected request for CGM, rejected request for an insulin pump, and follow-up as independent variables, with age and BMI as covariates. Rejected request for CGM/pump was based on participant self-reports of denials, without probing specific reasons (e.g., unavailability, insurance, or stock issues), as the focus was on associations with mental health outcomes.

Significance was set at *p* < 0.05. Given previous reports suggesting higher accuracy when choosing a higher cut-off on the PHQ-9 scale [23] and for higher specificity, we opted to choose the severe category for the predictive model to enhance clinical relevance and minimize misclassification bias inherent in self-reported data. Since the severe category is more likely to reflect true pathology, the identified risk factors are more likely to enhance detection of high-risk individuals.

## 3. Results

### 3.1. Demographic and Clinical Characteristics

A total of 1073 people with T1D (303 males, 770 females) participated in the study. Female patients were younger (25.6 vs. 28.3 years; *p* < 0.001), with a significantly lower BMI than male patients (24.1 vs. 25.1 kg/m^2^; *p* = 0.003). The proportion of obese patients was significantly higher among males than females (19.2% vs. 10.7%; *p* < 0.001). Overall, the prevalence of those who had T1D > 10 years was 53.5%, with a higher proportion of males having T1D < 10 years; more females had T1D > 10 years (*p* < 0.001). Female patients were more likely to have university degrees, whereas male patients were more likely to have postgraduate degrees (*p* = 0.17). Overall, only 22% of the respondents reported engaging in ≥150 min of physical activity per week, with the proportion of males being significantly higher than the proportion of females (29.4% vs. 19.4%; *p* < 0.001). Other comorbidities were more common in females than males, especially hypothyroidism (*p* = 0.008). No significant differences were observed in residence, income, or diabetes complications. Nearly half of the participants resided in the central region (48.0%), with similar distributions across males and females and no significant gender differences in regional residence (*p* = 0.88; Table 1).

### 3.2. T1D Management

There were no significant differences between males and females in the last HbA1c value reported, with most of the participants (66.9%) reporting HbA1c ≤ 8%. Moreover, no differences were observed in visits to the diabetes education clinic during the past 12 months, being counselled on carbohydrate counting, having medical insurance, or having a rejected request for continuous glucose monitoring (CGM) or an insulin pump (Table 2). Males were more likely than females to report an above-average benefit from visiting diabetes education clinics (35% vs. 28.7%; *p* = 0.03) or visiting a private hospital or local neighborhood clinic for diabetes care (20.5% vs. 13.1% and 7.3% vs. 3.4%, respectively; all *p* < 0.001). On the other hand, females were more likely than males to visit a clinical dietician (35.5% vs. 24.4%; *p* = 0.001), use an insulin pump for diabetes management (23.9% vs. 16.5%; *p* = 0.01), and practice carbohydrate counting (53% vs. 37%; *p* < 0.001) (Table 2).

### 3.3. Prevalence of Depression and Anxiety

**Figure 1:**
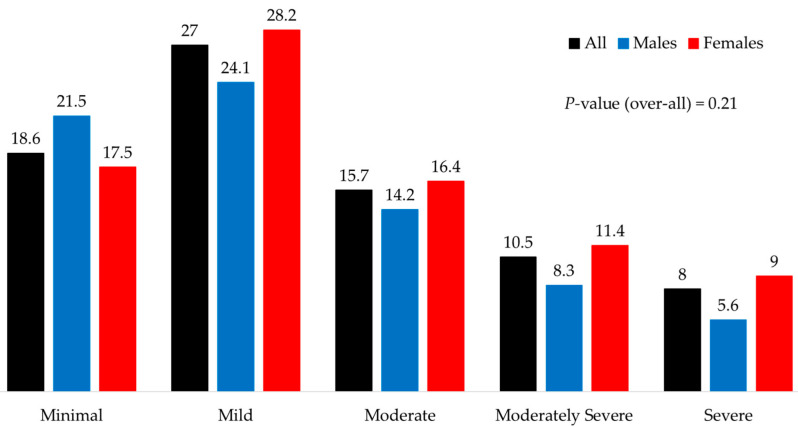
Prevalence of depression according to PHQ-9 scores in male and female patients with type 1 diabetes. Overall *p* = 0.201.

**Figure 2:**
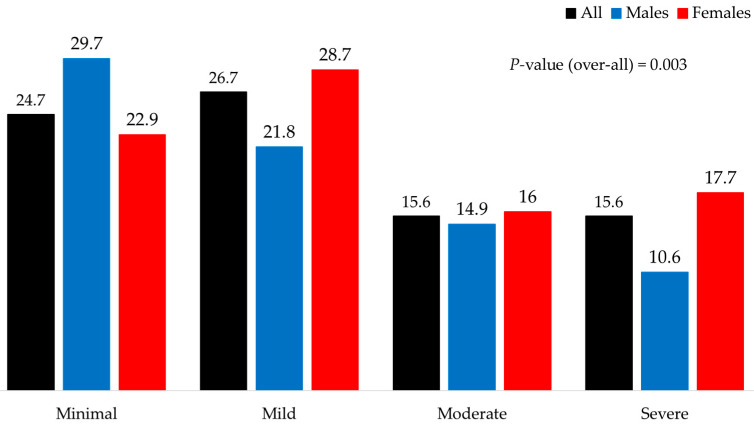
Prevalence of anxiety according to GAD-7 scores in male and female patients with type 1 diabetes. Overall *p* = 0.003.

The overall prevalence values of depression and anxiety stratified by gender with 95% confidence intervals are shown in Table 3. There was a modestly higher prevalence of moderate-to-severe depression in females compared to males (Figure 1), but this difference was not significant. A proportion of 61% of patients scored ≥5 on the PHQ-9 questionnaire, indicating mild or greater depression, with significantly more females included in this subgroup (64.9% vs. 52.1%; *p* = 0.02). Anxiety scores were higher among females (overall *p* = 0.003) (Figure 2), mainly due to the moderate-to-severe categories.

### 3.4. Depression Symptoms

Highlights of the PHQ-9 results are shown in Table 4. Overall, 18.5% of participants perceived moderately severe to severe depression, with no difference between males and females. There was a high prevalence of depressive symptoms among respondents; 28.9% reported little interest for ‘more than half the days’ to ‘nearly every day’, 30.6% reported feeling down for ‘more than half the days’ to ‘nearly every day’, and 42% reported fatigue and sleep disturbance issues at least ‘more than half the days’.

### 3.5. Anxiety Symptoms

High rates of perceived anxiety symptoms on the GAD-7 (Table 5) were also observed; 17.6% of respondents reported feeling nervous for ‘more than half the days’, whereas 17% reported feeling nervous ‘nearly every day’. Excessive worry, trouble relaxing, and irritability for ‘more than half the days’ was reported by 17% of respondents, and restlessness ‘nearly every day’ was reported by 15% of respondents.

### 3.6. Risk Factors of Perceived Severe Depression and Anxiety

Significant risk factors of perceived mental health disturbances in all respondents are shown in Table 6. People with T1D with a monthly income of 5000–10,000 SAR (odds ratio [OR] = 0.38, 95% confidence interval [CI] 0.20–0.75; *p* = 0.005) or 11,000–20,000 SAR (OR = 0.39, 95% CI 0.19–0.78; *p* = 0.008) had significantly lower odds of perceived severe depression than people with T1D with lower incomes. Absence of neuropathy (OR = 0.27, 95% CI 0.11–0.62; *p* = 0.002) and reporting an above-average benefit from diabetes education clinic visits (OR = 0.47, 95% CI 0.25–0.90; *p* = 0.02) were also significantly protective. Engaging in carbohydrate counting (OR = 0.47, 95% CI 0.30–0.75; *p* = 0.002) and having good glycemic control (i.e., HbA1c < 7.0%) substantially lowered the odds (OR = 0.24, 95% CI 0.08–0.72; *p* = 0.01). In contrast, those who relied on friends with T1D as a primary educational resource were more likely to experience perceived severe depression (OR = 2.8, 95% CI 1.1–7.2; *p* = 0.04). In addition, having a request for CGM rejected significantly increased the risk (OR = 1.88, 95% CI 1.1–3.2; *p* = 0.02), and having a request for an insulin pump rejected nearly tripled the odds of perceived severe depression (OR = 2.82, 95% CI 1.5–5.3; *p* = 0.001). “Rejected request” refers to participant-reported denials for CGM/insulin pumps without specified reasons (e.g., unavailability at site, insurance denial, or out of stock), potentially reflecting access barriers but limiting detailed interpretation.

For perceived severe anxiety, male patients were less likely to be affected than female patients (OR = 0.61, 95% CI 0.40–0.93; *p* = 0.02). The absence of neuropathy (OR = 0.38, 95% CI 0.18–0.80; *p* = 0.01) and practicing carbohydrate counting (OR = 0.54, 95% CI 0.38–0.76; *p* < 0.001) were also associated with a significantly reduced risk of perceived severe anxiety. Similarly, people with T1D with good glycemic control (i.e., HbA1c < 7.0%) had notably lower odds of perceived anxiety (OR = 0.24, 95% CI 0.10–0.58; *p* = 0.001). However, those who preferred self-directed learning from scientific sources had twice the risk of perceived severe anxiety (OR = 2.0, 95% CI 1.1–3.9; *p* = 0.03). Similar to perceived severe depression, rejection of an insulin pump request was a strong risk factor for severe anxiety, with affected people with T1D having more than twice the odds of experiencing it (OR = 2.36, 95% CI 1.45–3.79; *p* < 0.001).

## 4. Discussion

The present study provided a comprehensive overview of the demographic, clinical, psychosocial, and diabetes management characteristics of people with T1D in Saudi Arabia, highlighting key disparities between males and females and significant risk factors of mental health burden. To the best of our knowledge, the present study is the first to look into gender differences in T1D management on a national level with representation from all Saudi regions. Moreover, the study is the first to investigate the burden and risk factors of mental health and T1D on the national level.

Females constituted the majority of respondents and were significantly younger and had lower BMI than males. Despite better engagement in education-related behaviors, such as carbohydrate counting and dietitian visits, females demonstrated a higher burden of perceived psychological distress, particularly anxiety. Anxiety disorders are common mental health disorders that disproportionately affect women, with contributing factors such as differences in brain structure, genetic predisposition, and hormone fluctuations being commonly cited as underlying causes [14]. In the Saudi context, cultural gender roles—such as greater societal expectations for women to manage family responsibilities alongside chronic illness—may amplify this burden, leading to heightened anxiety despite proactive self-care [10,12,24]. This paradox suggests that while females excel in structured management, systemic barriers like limited autonomy in healthcare decisions or stigma around mental health seeking may undermine emotional well-being [25,26]. Studies on female T1D patients and mothers of T1D children show higher anxiety in caregivers due to emotional labor, aligning with our findings [9]. Many psychosocial factors have been observed to worsen anxiety in females, such as gender roles, expectations, and stigma [11,12]. Recent data from Saudi patients with diabetes reinforce female predominance in anxiety/depression [13,27]. To address this, routine use of validated questionnaires by healthcare workers should be reinforced. Gender-specific interventions could include women-only support groups, culturally sensitive provider training, and/or anonymous digital mental health platforms tailored to Saudi women with T1D.

The finding that females were more likely to score ≥5 on the PHQ-9, even without significant differences in severe depression, has important implications for clinical practice and policy. This threshold indicates at least mild depressive symptoms, which can progress to more severe forms if left unmanaged and potentially impair diabetes self-management, leading to poorer glycemic outcomes and increased complications [3,4]. In Saudi Arabia, where cultural stigmas around mental health may deter females from seeking help, this finding highlights the need for gender-sensitive approaches as discussed earlier. One key implication is an urgent need to improve access to psychiatry and psychological services for people with T1D by integrating mental health professionals into diabetes clinics to provide seamless, multidisciplinary care or, if not possible, to at least prioritize referrals from diabetes clinics for people with T1D. Implementing an annual questionnaire to screen for depression and anxiety using validated tools, such as the PHQ-9 and GAD-7, is within the bounds of standard diabetes care [2,28]. In Saudi Arabia, diabetes clinics need to be more attentive to this critical step while investigating reasons why such screening is not consistently implemented and allocating resources to overcome barriers. Potential barriers include resource constraints in public clinics, cultural stigma reducing patient disclosure (e.g., fear of judgment or family disapproval), limited awareness of mental health services, and lack of integrated electronic health records for routine screening, as seen in studies on diabetes care access and mental health help-seeking [29,30]. Practical integration could draw from models like collaborative care programs in the US or Europe, which embed psychologists in clinics for on-site counseling and shared decision-making, adapted to Saudi contexts through mobile apps or community-based outreach (e.g., via mosques or family centers) to address cultural sensitivities and stigma [29]. This attention is warranted, as depressive disorders and diabetes rank among the top burdens of disease in the country, with mental disorders accounting for approximately 7.3% of total disability-adjusted life years and ranking as the 4th leading cause of disability [31].

In contrast, males had higher obesity rates and were more likely to lack regular follow-up or rely on injections rather than insulin pumps. The gender differences in cardiometabolic risks observed in the present study reinforce gender disparities in T1D self-care [32]. Although similar patterns have been observed in other countries [33], gender differences are not universally consistent, suggesting that such disparities are influenced by both biological factors and the degree of gender equality reflected in societal roles [25,26]. Overall, poor physical activity levels were reported, with only 22% of respondents meeting recommended guidelines, underscoring a significant behavioral gap in T1D self-care in Saudi Arabia.

The observed prevalence of psychological symptoms are in accordance with similar studies in the region [27,34,35]. However, key differences emerge: Mahmoud et al. [27] examined a mixed T1D/T2D population in Saudi Arabia and Egypt and reported higher anxiety prevalence in Egypt (40%), likely influenced by socioeconomic disparities. In contrast, the present study focused on Saudi individuals with T1D and uniquely highlights technology denial (e.g., CGM/pump rejection) as a significant risk factor, an aspect which was absent in their work. Similarly, in Kuwait, AlOzairi et al. [34] emphasized distress predictors like age/education but not gender-specific rejection barriers. Compared to Younes et al. [35], who conducted a study in the UAE (high distress in adolescents), the present sample showed a persistent gender gap into adulthood, suggesting that cultural stigma may exacerbate under-reporting in Saudi males [10]. These comparisons underscore our study’s contribution to T1D-specific, national-level insights in Saudi Arabia while aligning with regional and global patterns of female vulnerability.

Globally, depression prevalence in people with T1D ranges from 22 to 30% [5,6], aligning with our findings but higher than in high-income Western countries (e.g., 15–20% in the US T1D Exchange [32]). Anxiety rates observed in the present study (moderate–severe in ~30%) exceed global averages (~20%) [5], potentially due to unique Middle Eastern factors like lifestyle, limited technological access, delayed diagnosis and presentation, and cultural stigma. Western studies have emphasized biological predictors [3,4], while the present results uniquely highlight socioeconomic and healthcare access barriers (e.g., technology denial) as key drivers in Saudi Arabia, underscoring the need for context-specific policies. Comparison with non-T1D populations shows similar gender patterns, with females reporting higher anxiety due to psychosocial factors, but T1D, as a chronic disease, adds illness-specific burdens like but not limited to caregiver stress [9,11,12].

Multivariable regression revealed protective factors against severe depression and anxiety, including higher income, the absence of neuropathy, good glycemic control (HbA1c < 7.0%), and structured self-care behaviors such as carbohydrate counting and perceived benefit from diabetes education. A notable finding in the present study is that denial of technology, particularly the refusal of insulin pumps or CGM, was strongly linked to perceived severe psychological distress, highlighting that the adoption and sustained use of technologies in T1D management has its own set of psychological challenges, independent of glycemic control [36]. The available literature suggests a clear link between CGM metrics such as repeated hypoglycemia and increased anxiety, whereas better glucose control is associated with lower anxiety levels, suggesting that the rejection of an insulin pump or CGM device may create a negative feedback loop in which patients feel compelled to exert more effort to manage their condition without the aid of supportive technology [37,38]. These predictors offer insights into systemic issues such as reliance on friends with T1D or self-directed reading as primary education sources (OR = 2.8 and 2.0, respectively), suggesting low trust and/or limited accessibility to formal healthcare systems, forcing people with T1D into informal networks that lack evidence-based guidance and potentially perpetuate misinformation or individualized experience [39,40,41]. This could also reflect broader barriers in Saudi healthcare, where socioeconomic factors and technology denial hinder professional engagement. Similarly, rejection of CGM or insulin pump requests (OR = 1.88–2.82) highlights how denied access to technology not only may affect glycemic control but also patient trust in the system, fostering feelings of helplessness and distress. Longitudinal studies exploring rejection reasons for policy refinement are recommended to confirm associations, as well as further investigation on the psychological burden associated with technology use in people with T1D.

Healthcare delivery is already a concern among Saudis with diabetes and severe mental illness [29]. Although the core psychosocial barriers may be similar across the world [39,40,41], their manifestation and drivers are shaped by cultural, social, economic, and healthcare contexts. Among the notable findings supporting this concern in the present study is dependence on peer-led or self-directed education rather than professional guidance, which was also associated with increased psychological burden, reflecting structural barriers that Saudi people with T1D face in the local healthcare system, consequently leading to additional psychological burden. The findings suggest issues on medical care access, whether for education, assessment, or obtaining tools to ease the burden of T1D self-care (e.g., CGM or insulin pump), eventually affecting patient trust in the system and possibly leading individuals to seek alternative sources (in this case, peer advice for diabetes education). Widening the gap between patients and the healthcare system will ultimately lead to late presentation of diseases such as mental health issues [12,17,35,36,37]. To mitigate this, policies should prioritize increased and equitable access to diabetes technologies, trust-building initiatives (e.g., patient-centered education programs), and integrated mental health screening to close the gap and prevent the exacerbation of psychological distress, strengthening overall public trust in the healthcare system.

The present study’s strengths include its large, geographically diverse sample and its focus on under-represented Saudi people with T1D, integrating demographic, clinical, behavioral, and mental health data. Standardized tools and adjusted regression models enhance the validity of the findings. However, several limitations must also be acknowledged. The cross-sectional design limits causal inference, and the self-reported nature of both clinical and psychological measures may introduce bias. Given the cross-sectional design and not knowing the reasons behind “rejected requests” for CGM/insulin pumps (self-reported), the findings should be considered associations, not causal. Selection bias is also possible due to the online survey methodology, possibly excluding older or digitally disconnected individuals. For clinical variables like HbA1c, comorbidities, and CGM/insulin pump rejections, recall bias could affect accuracy, as these were not validated against medical records—a common challenge in large-scale surveys [11,23]. Future studies should incorporate record linkage to enhance reliability. Gender differences in self-reporting may exacerbate bias, with males under-reporting [10]. Furthermore, despite adjustments for BMI and age, residual confounding cannot be ruled out. The true prevalence of severe depression and anxiety among Saudi people with T1D needs to be verified clinically. Lastly, the use of a convenience snowball sampling method without controlling for regional population distribution raises concerns about representativeness, particularly as nearly half of the cohort was from the central region (Riyadh and Al Qassim regions). While this aligns with the region’s demographic weight (~30.7% of the Saudi population; Saudi General Authority for Statistics, 2022 Census) [22] and T1D incidence patterns [21], it may under-represent other less populated regions. Future studies should use stratified or probability sampling to ensure balanced regional coverage and more solid conclusions.

## 5. Conclusions

People with T1D in Saudi Arabia experience high levels of perceived depression and anxiety, with notable gender differences and critical gaps in T1D management behaviors. Denial of new available technology aiding in T1D management and inadequate access to diabetes education appear to exacerbate the mental health risks. These findings underscore the need for integrated care models that not only promote metabolic control but prioritize mental health support, equitable and widespread access to diabetes technologies, and culturally tailored education. Policymakers should consider expanding insurance coverage, improving access to psychological services, and embedding mental health screening within T1D clinics to ensure holistic care for Saudi people with T1D.

## Figures and Tables

**Table 1:** Demographic characteristics of the cohort (*n* = 1073).

Characteristics	All	Males (*n* = 303)	Females (*n* = 770)	*p*-Value
Age (years)	26.4 ± 8.9	28.3 ± 11.1	25.6 ± 6.5	<0.001
BMI (kg/m^2^)	24.4 ± 5.5	25.1 ± 6.5	24.1 ± 5.0	0.003
Obesity status				<0.001
Underweight	175 (16.9)	54 (18.8)	121 (16.2)	
Normal Weight	461 (44.5)	107 (37.3)	354 (47.3)	
Overweight	264 (25.5)	71 (24.7)	193 (25.8)	
Obese	135 (13.0)	55 (19.2)	80 (10.7)	
Diabetes duration				<0.001
<1 year	83 (7.7)	31 (10.2)	52 (6.8)	
1–5 years	233 (21.7)	79 (26.1)	154 (20)	
6–10 years	183 (17.1)	61 (20.1)	122 (15.8)	
>10 years	574 (53.5)	132 (43.6)	442 (57.4)	
Residence				0.88
Central	515 (48.0)	141 (46.5)	374 (48.6)	
West	289 (27.0)	85 (28.1)	204 (26.5)	
East	127 (11.8)	39 (12.9)	88 (11.4)	
North	65 (6.1)	16 (5.3)	49 (6.4)	
South	76 (7.1)	22 (7.3)	54 (7.0)	
Education				0.17
High school	345 (32.2)	107 (35.3)	238 (30.9)	
University	646 (60.2)	169 (55.8)	477 (61.9)	
Postgraduate	82 (7.6)	27 (8.9)	55 (7.1)	
Physical activity (min/week)	97.8 ± 129.6	122.1 ± 155.3	88.2 ± 116.7	<0.001
Physical activity				<0.001
<150 min/week	833 (77.8)	214 (70.6)	619 (80.6)	
≥150 min/week	238 (22.2)	89 (29.4)	149 (19.4)	
Income				0.19
<5000 SAR	197 (18.4)	60 (19.8)	137 (17.8)	
5000–10,000 SAR	293 (27.3)	76 (25.1)	217 (28.2)	
11,000–20,000 SAR	247 (23.0)	78 (25.7)	169 (21.9)	
21,000–30,000 SAR	65 (6.1)	21 (6.9)	44 (5.7)	
31,000–40,000 SAR	38 (3.5)	11 (3.6)	27 (3.5)	
>40,000 SAR	37 (3.4)	14 (4.6)	23 (3.0)	
I don’t know	196 (18.3)	43 (14.2)	153 (19.9)	
Diabetes complications				0.11
None	724 (71.0)	194 (69.5)	530 (71.5)	
I don’t know	133 (13.0)	35 (12.5)	98 (13.2)	
Neuropathy	44 (4.3)	12 (4.3)	32 (4.3)	
Nephropathy	29 (2.8)	7 (2.5)	22 (3.0)	
Retinopathy	80 (7.5)	25 (9.0)	55 (7.4)	
Diabetic foot	4 (0.4)	1 (0.4)	3 (0.4)	
Coronary artery disease	6 (0.6)	5 (1.8)	1 (0.1)	
Other comorbidities				0.008
None	866 (82.6)	265 (88.6)	601 (80.1)	
Hypothyroidism	121 (11.5)	18 (6.0)	103 (13.7)	
Celiac disease	49 (4.7)	12 (4.0)	37 (4.9)	
Vitiligo	11 (1.0)	3 (1.0)	8 (1.1)	
Addison’s disease	2 (0.2)	1 (0.3)	1 (0.1)	

Note: Data are presented as mean ± standard deviation or *n* (valid %). Significance set at *p* < 0.05. BMI, body mass index.

**Table 2:** T1D management in the cohort (*n* = 1073).

Parameter	All	Males (*n* = 303)	Females (*n* = 770)	*p*-Value
Last known HbA1c				0.10
<7.0%	388 (36.2)	103 (34.0)	285 (37.0)	
7.1–8.0%	329 (30.7)	86 (28.4)	243 (31.6)	
8.1–9.0%	151 (14.1)	45 (14.9)	106 (13.8)	
>9.0%	163 (15.2)	50 (16.5)	113 (14.7)	
I don’t know	42 (3.9)	19 (6.3)	23 (3.0)	
Visited diabetes education clinic last year	714 (66.5)	199 (65.7)	515 (66.9)	0.72
Benefit from diabetes education clinic visit				0.03
Above average	327 (30.5)	106 (35.0)	221 (28.7)	
Average	277 (25.8)	61 (20.1)	216 (28.1)	
Less than average	247 (23.0)	67 (22.1)	180 (23.4)	
No benefit	222 (20.7)	39 (22.8)	153 (19.9)	
Education resource preferences				0.04
Diabetes education clinic	90 (8.4)	35 (11.9)	55 (7.3)	
Attending physician	123 (11.7)	43 (14.6)	80 (10.6)	
Diabetes groups on social media	75 (7.1)	23 (7.8)	52 (6.9)	
Friends with diabetes	40 (3.8)	11 (3.7)	29 (3.8)	
Own reading of scientific sources	69 (6.6)	20 (6.8)	49 (6.5)	
Multiple education sources	655 (62.3)	163 (55.3)	492 (65.0)	
Advised carbohydrate counting				0.14
Yes, but no referral to dietician	244 (22.7)	64 (21.1)	180 (23.4)	
Yes, and referred to dietician	565 (52.7)	152 (50.2)	413 (53.6)	
No	264 (24.6)	87 (28.7)	177 (23.0)	
Practices carbohydrate counting	520 (48.5)	112 (37.0)	408 (53.0)	<0.001
Visited dietician clinic	347 (32.3)	74 (24.4)	273 (35.5)	0.001
Has medical insurance	321 (30.0)	113 (37.3)	208 (37.2)	0.91
Rejected request for CGM	382 (46.8)	104 (47.9)	278 (46.4)	0.38
Rejected request for insulin pump	327 (51.8)	80 (48.8)	247 (52.9)	0.210
Type of clinic visited for follow-ups				<0.001
Government hospital	696 (64.9)	173 (57.1)	523 (67.9)	
Private hospital	163 (15.2)	62 (20.5)	101 (13.1)	
Local clinic	48 (4.5)	22 (7.3)	26 (3.4)	
I don’t have regular follow-ups	125 (11.6)	11 (3.6)	30 (3.9)	
Type of management				0.01
Insulin injection	839 (78.2)	253 (83.5)	586 (76.1)	
Insulin pump	234 (21.8)	50 (16.5)	184 (23.9)	
PHQ-9 score ≥ 5	658 (61.2)	158 (52.1)	500 (64.9)	0.02

Note: Data are presented as *n* (valid %). Significance set at *p* < 0.05. CGM, continuous glucose monitoring.

**Table 3:** Prevalence of depression and anxiety.

	Overall	Males	Females
Depression			
Minimal	18.6 (16.3–20.9)	21.5 (16.8–26.1)	17.5 (14.8–20.2)
Mild	27 (24.3–29.6)	24.1 (19.2–28.9)	28.2 (25.0–31.4)
Moderate	15.7 (13.5–17.9)	14.2 (10.2–18.1)	16.4 (13.7–19.0)
Moderately Severe	10.5 (8.7–12.4)	8.3 (5.1–11.4)	11.4 (9.2–13.7)
Severe	8 (6.4–9.6)	5.6 (3.0–8.2)	9 (6.9–11.0)
Anxiety			
Minimal	24.7 (22.1–27.3)	29.7 (24.5–34.9)	22.9 (19.9–25.8)
Mild	26.7 (24.0–29.4)	21.8 (17.1–26.5)	28.7 (25.5–31.9)
Moderate	15.6 (13.4–17.8)	14.9 (10.8–18.9)	16 (13.4–18.6)
Severe	15.6 (13.4–17.8)	10.6 (7.1–14.0)	17.7 (15.0–20.4)

Note: Data presented as % (95% confidence interval).

**Table 4:** PHQ-9 responses [Over the last 2 weeks, how often have you been bothered by any of the following problems?].

Question	Not at All	Several Days	More Than Half the Days	Nearly Every Day
Little interest or pleasure in doing things?	267 (29.6)	374 (41.5)	127 (14.1)	133 (14.8)
Feeling down, depressed, or hopeless?	220 (24.4)	405 (45.0)	156 (17.3)	120 (13.3)
Trouble falling or staying asleep, or sleeping too much?	233 (25.9)	300 (33.3)	173 (19.2)	195 (21.6)
Feeling tired or having little energy?	125 (13.9)	398 (44.2)	180 (20.0)	198 (22.0)
Poor appetite or overeating?	226 (25.1)	314 (34.9)	180 (20.0)	181 (20.1)
Feeling bad about yourself?	459 (50.9)	222 (24.6)	87 (9.7)	133 (14.8)
Trouble concentrating on things?	444 (49.3)	265 (29.4)	107 (11.9)	85 (9.4)
Moving/speaking slowly, or the opposite?	568 (63.0)	197 (21.9)	73 (8.1)	63 (7.0)
Thoughts that you would be better off dead?	687 (76.2)	119 (11.1)	44 (4.9)	51 (5.7)

Note: Data are presented as *n* (valid %).

**Table 5:** GAD-7 responses [Over the last 2 weeks, how often have you been bothered by any of the following problems?].

Question	Not at All	Several Days	More Than Half the Days	Nearly Every Day
Feeling nervous, anxious, or on edge?	113 (12.7)	399 (44.9)	189 (17.6)	188 (17.5)
Not being able to stop or control worrying?	242 (22.5)	326 (30.3)	151 (17.0)	170 (19.1)
Worrying too much about different things?	171 (19.2)	353 (39.7)	166 (18.7)	199 (22.4)
Trouble relaxing?	270 (30.4)	327 (36.8)	159 (17.9)	133 (15.0)
Being so restless that it is hard to sit still?	489 (55.0)	235 (26.4)	90 (10.1)	75 (8.4)
Becoming easily annoyed or irritable?	170 (19.1)	343 (38.6)	161 (18.1)	215 (24.2)
Feeling afraid, as if something awful might happen?	431 (48.5)	239 (26.9)	97 (10.9)	122 (13.7)

Note: Data are presented as *n* (valid %).

**Table 6:** Significant risk factors of severe depression and anxiety.

Severe Depression			
Factor	Odds Ratio	Confidence Interval	*p*-Value
Income			
5000–10,000 SAR	0.38	0.20–0.75	0.005
11,000–20,000 SAR	0.39	0.19–0.78	0.008
Diabetes complications			
Absence of neuropathy	0.27	0.11–0.62	0.002
Benefit from diabetes education clinic visit			
Above average	0.47	0.25–0.90	0.02
Education resource preferences			
Friends with T1D	2.8	1.1–7.2	0.04
Practices carbohydrate counting	0.47	0.30–0.75	0.002
Last known HbA1c			
<7.0%	0.24	0.08–0.72	0.01
Rejected request for CGM	1.88	1.1–3.2	0.02
Rejected request for insulin pump	2.82	1.5–5.3	0.001
Severe Anxiety			
Gender			
Male	0.61	0.40–0.93	0.02
Diabetes complications			
Absence of neuropathy	0.38	0.18–0.80	0.01
Education resource preferences			
Own reading of scientific sources	2.0	1.1–3.9	0.03
Practices carbohydrate counting	0.54	0.38–0.76	<0.001
Last known HbA1c			
<7.0%	0.24	0.10–0.58	0.001
Rejected request for insulin pump	2.36	1.45–3.79	<0.001

Note: All *p*-values were adjusted for age and body mass index. CGM, continuous glucose monitoring; T1D, type 1 diabetes.

## Data Availability

The data that support the findings of this study are available upon request from the corresponding author. The data are not publicly available due to privacy or ethical restrictions.
